# Managing urinary incontinence: evaluating the role of practice facilitators in enhancing quality improvement and digital integration in primary care

**DOI:** 10.1186/s43058-026-00936-9

**Published:** 2026-05-08

**Authors:** Nipher Malika, Shona Olalere Oluwatola, Gabriela Alvarado, Sangeeta Ahluwalia

**Affiliations:** 1 RAND, 1776 Main St., Santa Monica, CA USA; 2 RAND, 1200 S Hayes St., Arlington, VA USA

**Keywords:** Practice facilitation, Urinary incontinence, Quality improvement, Primary care, Implementation barriers, Sustainability

## Abstract

**Background:**

Practice facilitation is an established strategy for advancing quality improvement in primary care, particularly in chronic disease management. However, knowledge gaps remain regarding its long-term impact, integration of digital tools (e.g., telehealth), and barriers encountered during implementation. This study aimed to address these gaps by evaluating the Agency for Healthcare Research and Quality (AHRQ)-funded Managing Urinary Incontinence (MUI) Initiative, which supported five US institutions in implementing evidence-based, nonsurgical treatments for urinary incontinence (UI) in women.

**Methods:**

Over a two-year period, we conducted virtual semi-structured interviews and focus groups with 15 practice facilitators from each grantee institution, all of whom worked with a total of 270 practices. Discussions explored their evolving roles, implementation processes, challenges encountered, and strategies for supporting UI care. Data were audio-recorded, transcribed, and subjected to thematic analysis in Dedoose, using a team-developed codebook with dual independent coders.

**Results:**

Five major themes emerged: (1) practice facilitator role conceptualization varied across settings and evolved over time; (2) facilitators were central to supporting process improvements with tailored resources; (3) technology integration, such as telehealth and electronic health records (EHRs), was increasingly leveraged but unevenly adopted; (4) substantial barriers included navigating institutional bureaucracy and securing physician champions amidst competing demands; and (5) facilitators implemented strategies for sustaining practice change beyond the funded intervention. Despite successes, facilitators struggled with limitations of time, resources, and systemic constraints.

**Conclusions:**

The findings provide actionable insights into the facilitation process for implementing evidence-based UI care, highlighting the importance of adaptive facilitator roles, technology use, and strategies for overcoming barriers and sustaining improvements. This research contributes to practical approaches for implementation in primary care settings.

**Supplementary Information:**

The online version contains supplementary material available at 10.1186/s43058-026-00936-9.

Contributions to the literature

- This study explores how practice facilitators adapt their roles over time to address challenges in implementing evidence-based care for urinary incontinence in primary care.
- Highlights practical strategies for integrating technology, such as telehealth and digital tools (electronic health records, patient portals, mobile health applications, digital communication platforms such as Microsoft Teams, tele-education platforms like Project ECHO, and data dashboards), into facilitation efforts to improve care processes.
- Identifies key barriers to practice facilitation, including organizational bureaucracy and engaging clinical champions, and offers solutions for overcoming them.
- Provides new insights into sustaining quality improvement after external support ends, informing future implementation efforts for under-addressed health issues.

## Introduction

Primary care is increasingly tasked with delivering evidence-based care aligned with value-based models, patient-centered outcomes, and cost-effectiveness [1–5]. Practice facilitation is a key strategy to support these practices in achieving their quality improvement goals [5–10]. This approach involves deploying trained professionals, often called practice facilitators or practice coaches, who work collaboratively with healthcare teams to enhance their capacity for implementing changes and improving overall care delivery [11–14]. Effective practice facilitators significantly strengthen primary care settings, making practices and physicians nearly three times more likely to adopt evidence-based guidelines [8, 15, 16]. Several studies have also shown that practice facilitation significantly impacts the prevention, treatment, and management of chronic diseases [9, 15–19]. Additionally, facilitators have played a crucial role in integrating community health workers into care teams, supporting community–clinical linkage models and enhancing healthcare delivery across diverse settings [7, 13, 20].

Despite strong evidence of effectiveness—demonstrating that practice facilitation alone can improve care process measures across chronic diseases, particularly in under-resourced, small, independent practices where quality infrastructure is weakest [5, 15, 16]— several gaps remain. Longitudinal studies of practice facilitation are scarce [8, 21], and there is limited understanding of how facilitators are selected, and supported, as well as how their roles vary across intervention contexts [22]. Persistent implementation barriers—ranging from workforce burnout and role ambiguity to system-level bureaucracy and sustainability challenges after the withdrawal of external support—are often underexplored, and few qualitative studies have captured the nuanced, real-world adaptations and challenges faced by facilitators themselves [23, 24].

These persistent implementation questions are especially pressing for high-burden, under-addressed conditions in primary care. Urinary incontinence (UI) is a prime example: in the U.S., approximately 30% of older women and more than 50% of all women report UI—a condition strongly linked to depression and disability, yet it remains inadequately identified, managed, or prioritized in healthcare settings, despite the availability of evidence-based solutions [25–27]. UI is considered under-addressed because it is frequently overlooked during primary care visits, rarely discussed due to stigma and competing priorities, and systematic screening or management is often lacking despite its prevalence and impact [27, 28]. This occurs even though established clinical guidelines—such as those from the American College of Physicians and the American Urological Association—recommend routine screening, patient education, lifestyle interventions, pelvic floor muscle training, and referral when indicated as standard components of UI care [27, 29]. This pattern highlights a striking gap between best practices and actual clinical care and underscores the critical need for implementation strategies that can bridge this divide in everyday practice. Notably, to our knowledge, no prior studies have utilized practice facilitators to address UI in primary care.

Thus, to address these gaps, the Agency for Healthcare Research and Quality (AHRQ) launched the Managing Urinary Incontinence (MUI) Initiative in 2021.

### AHRQ MUI grantees

The AHRQ MUI Initiative sought to improve the identification and management of UI among women in primary care through three-year grants awarded to five diverse institutions nationwide. Each grantee implemented tailored, evidence-based strategies—leveraging practice facilitators as a core component—to address care gaps across various clinical and community contexts, including large academic health systems, urban networks, rural and tribal clinics, and federally qualified health centers. Practice facilitators drove clinic engagement, workflow redesign, staff education, technology integration, and ongoing process improvement, adapting their approaches to local needs. Collectively, this multi-site initiative generated practical insights into overcoming barriers and implementing evidence-based UI care in real-world settings. These grantees were:

- **IT2 (Illinois)**targeted UI screening and management in a large academic health system, integrating automated screening within the patient portal and embedding decision support and shared decision-making tools in the electronic health record (EHR) to promote consistent, guideline-based care [30].
- **OPTIMA (California)**worked across four major health systems that serve highly diverse populations. This provider-focused project used academic detailing, EHR-linked decision support, advanced practice provider co-management, and electronic referral pathways to enhance quality improvement in UI care delivery [30].
- **WI-INTUIT (Wisconsin)**operated in varied primary care environments—including large health systems, federally qualified health centers (FQHCs), tribal clinics, and independent practices—adopting streamlined practice facilitation and leveraging community partnerships to adapt and implement UI screening and referral processes, specifically targeting rural and underserved populations defined by HRSA/FQHC guidelines [30, 31].
- **EMPOWER (Ohio)**implemented a patient-centered intervention within an academic health system, combining nurse navigation, a mobile AI-driven chatbot, and Project ECHO tele-education for providers to encourage self-management and address barriers to accessing UI care [30].
- **PURSUIT (Alabama, Georgia, South Carolina)**worked within Veterans Health Administration (VHA) community clinics, focusing on female veterans. This project employed a mobile health app, an extensive online toolkit, and structured practice facilitation to expand access to nonsurgical, evidence-based UI treatments [30].

### Study aim

This study sought to identify and describe the various approaches to practice facilitation that could enhance the management of UI, thereby contributing to the integration of evidence-based practices in primary care and improved patient outcomes. By synthesizing qualitative insights from practice facilitators operating in distinct organizational and community contexts, this work seeks to contribute to our understanding of practice facilitation for under-addressed conditions in primary care and inform future implementation efforts.

## Methods

### Study design and procedures

We carried out a series of semi-structured interviews lasting 30–60 min with each grantee’s practice facilitator(s) via Microsoft Teams twice: first, one year after the project launch (*n* = 13), and a second time in the third year of the project (*n* = 11). Practice facilitators from each grantee project were invited via email to participate in the interviews and were informed that the interviewers represented an independent, third-party evaluation team funded to assess the projects. Across the five grantee sites, these practice facilitators worked with 270 practices and 844 providers as part of the MUI initiative.

In the first year of implementation, we conducted four interviews (*n* = 8) and one focus group (*n* = 5) with practice facilitators. In the second year, we conducted five additional interviews with most of the same facilitators (*n*= 11); five were unavailable due to scheduling conflicts. Some key informant interviews included multiple key informants, but none exceeded four participants, in line with qualitative best practices [32–34]. Interview questions addressed the implementation and evolution of facilitator roles, their backgrounds, and the challenges and supports encountered in helping practices adopt evidence-based UI care. In year two, additional prompts explored changes in facilitator responsibilities and plans for sustaining practice changes. See Table 1 for sample interview questions. The reporting of this work was guided by the COREQ Checklist (Supplemental 1).

**Table 1:** Sample interview questions

BACKGROUND Year 1 1) How did you come to this role? What does your background and prior experience look like and how did that prepare you for this role? 2) How would you define the role “practice facilitator” in [Grantee project name]? What’s the purpose of the practice facilitator(s) in the project? What does(do) the practice facilitator(s) do? Year 2 3) How has practice facilitator role changed or evolved since we last talked in [month/year]?
REACH Year 1 1) What were (or have been) the biggest challenges in recruiting primary care practices for [Grantee project name]? 2) What strategies are you finding most effective for recruiting primary care practices? Year 2 3) If PFs are involved in recruitment, how has that changed from the last time we talked with them in (month/year)? 4) What were (or have been) the biggest challenges in recruiting primary care practices for [Grantee project name]?
PROCESS Year 1 1) What types of technical assistance, resources, and support do you provide to primary care practices in [Grantee project name]? 2) What are the main *challenges* you have faced in engaging and supporting practices? (or what anticipated challenges do you foresee in engaging and supporting practices?) Year 2 3) How do the types of assistance and resources you provide differ among primary care practices that you support, and why? Has this changed from the start of the project to now? 4) How is the support varied or tailored for different types of practices (e.g., rural vs urban, large vs small), their capacity for practice improvement, or their UI care for women at their start of the project?
IMPACT Year 1 1) To you, what defines (will define) a practice as a successful participant in [Grant project name]? 2) What can practices do now to help ensure that successful changes and improvements to UI care will be sustained? Year 2 3) How much longer will you, as practice facilitators, expect to keep working with the practices participating in [Grantee project name]? 4) What are things you as a practice facilitator have been doing to ensure sustainability and what are the practices doing?

Two female PhD policy researchers (authors NM and GA) conducted the interviews in the spring of 2023 and summer of 2024. The interviewers are trained in qualitative methods in behavioral science and health services research and possess extensive prior experience interviewing and analyzing qualitative data. All interviews were recorded and transcribed verbatim. This qualitative study received ethical approval from RAND’s Human Subjects Protection Committee, and all participants provided verbal informed consent.

### Data analysis

We employed thematic analysis as it enabled us to systematically identify and interpret recurring patterns and themes across the five grantee projects, offering detailed insights into their approaches to practice facilitation [35]. Due to the small sample number of grantee projects evaluated (*n*= 5) and only 16 practice facilitators, data were deidentified to ensure confidentiality. Transcripts were then cleaned and uploaded to Dedoose (a secure, web-based qualitative and mixed-methods analysis software) for qualitative coding [36]. A codebook was developed by one author (SO) and finalized by the team through an iterative approach utilizing inductive codes. The team iterated and restructured the codebook as new findings emerged. Two independent researchers (SO, NM) double coded the transcripts and thematic analysis occurred with the whole study team to identify patterns across the coded data regarding practice facilitation [37, 38]. The team’s inter-rater reliability was 0.78, a high level of reliability [39]. The study team convened to collectively discuss and summarize findings within and across themes. We define themes as short sentences or phrases that summarize or categorize a recurring pattern that we observed in the coded data.

## Results

### Background and roles of practice facilitators

Facilitators were selected for grantee projects for their unique insights (e.g., experience with the health care system, prior experience as a practice facilitator) and expertise (e.g., nursing, psychology, involvement with practices) to enhance the facilitation process. Each grantee pursued distinct expertise based on their envisioned role for the practice facilitator. Some desired individuals with health project management experience, while others preferred those with clinical backgrounds to better connect with the clinicians involved. Additionally, some grantees opted for multiple facilitators to encompass clinical expertise, community-level experience with practices, and project management skills. Among the projects, only one offered formal training for practice facilitators, while the others primarily relied on experiential, on-the-job learning.

The number of facilitators ranged from one to five (*n* = 15) with varying backgrounds, roles, targeted areas, and activities (See Table 2); majority had never served as a practice facilitator nor had any awareness of the practices they would be engaging in. Of the practice facilitators interviewed, 11 also served as project or research managers or had a research background that qualified them for the role. The group also included a urologist, a urogynecologist, a geriatric physician, a nurse practitioner, and a researcher. While practice facilitators are typically trained professionals who support healthcare practices in implementing evidence-based improvements, in this UI initiative, some clinicians also took on these responsibilities. Their direct clinical experience and familiarity with the challenges faced by practices allowed them to effectively support and guide improvement efforts.

**Table 2:** Practice Facilitator Background, Roles and Facilitation Implementation

**MUI Grantee**	**# of Grantee Facilitators**	**Facilitator Backgrounds & Role in Grant Project**	**Targeted Components of Primary Care**	**Facilitation Aims and Activities**
**Grantee 1**	1	• Facilitator(s) Background: Dedicated practice facilitator with project management roles and clinical experience • Role in project: Clinical quality, working with analysts on data assessment, practice engagement, facilitation and orientation	• Practice leaders • Intervention team • Care processes	• Recruitment/ Practice Engagement • Improve Healthcare Quality
**Grantee 2**	3	• Facilitator(s) Background: 2 Urology clinicians and 1 project manager with project management roles, study research experience, • Role in project: Facilitation for providers will be the UI and primary care specialist; they will provide academic detailing; Each site leader for each health system will have one person facilitating at the system level	• Providers • Health system individual enrolled providers	• Recruitment/ Practice Engagement • Improve Healthcare Quality • Promote Provider Satisfaction • Facilitate Organizational Change
**Grantee 3**	5	• Facilitator(s) Background: 3 research/project managers, 1 urogynecologist, 1 researcher, managers with management roles, study research experience, and clinical experience • Role in project: Engagement with clinics to determine and facilitate best fit intervention workflow, engagement of clinical staff, provision of educational training and resources	• Practice leaders • Intervention team • Care processes	• Recruitment/ Practice Engagement • Improve Healthcare Quality • Fostering Community Partnerships • Resource Provision
**Grantee 4**	2	• Facilitator(s) Background: Dedicated practice facilitator with project/research management roles, study research experience, and clinical experience • Role in project: Practice facilitation to align with intervention workflow, engagement of clinical staff, provision of educational training	• Practice leaders • Intervention team • Care processes • Providers	• Recruitment/ Practice Engagement • Improve Healthcare Quality • Increase Practice Efficiency • Provide Educational Opportunities
**Grantee 5**	4	• Facilitator(s) Background: 1 nurse practitioner, 3 project/research managers with project management roles, study research experience, and clinical experience • Role in project: engagement with clinicians, clinician and practice engagement, champions	• Practice leaders • Intervention team • Care processes	• Recruitment/ Practice Engagement • Improve Healthcare Quality • Increase Practice Efficiency • Resource Provision

Over the two-year data collection period, we observed notable changes in practice facilitation. In Year 1, facilitators were initially uncertain and focused on determining how best to engage and support their practices. By Year 2, however, they had developed greater confidence and established stronger rapport with their practices, resulting in more effective facilitation.

We identified five themes from the qualitative interview and focus groups: 1) varying conceptualization of practice facilitator role; 2) supporting process improvement in clinic settings; 3) leveraging technology to facilitate change in healthcare practice; 4) barriers to practice facilitation; and 5) sustainable practice changes beyond practice facilitation. Below, we describe each theme, as well as provide illustrative quotes to exemplify with more detail and nuance.

### Theme 1: Varying conceptualization of practice facilitation role

In year one, as each grantee project had a unique structure and design, the practice facilitation conceptualization and approaches varied greatly. One grantee team began by utilizing the ARHQ practice facilitation handbook [40] and then tailored the practice facilitator role to alleviate the burden on each individual practice.

“We began with the ARHQ Handbook on practice facilitation, [highlighted] all of those major components [then decided to] see how much burden, we shift from the practices to the practice facilitation team knowing that down the line and sustainability and scalability there may be further opportunities to reduce even that burden.” - Grantee 3, interviewee 1

Three of the grantees in year one, viewed practice facilitators as responsible for recruiting practices and maintaining ongoing communication with practice sites (through emails, Microsoft team, phone calls and in-person site visits). Very often this was the role clinicians played. One of the three grantees also required their facilitator to identify innovative strategies for keeping practices engaged. While another grantee’s practice facilitation effort focused on engagement with the physicians rather than the practices themselves.

“The facilitator is the person kind of going out there and recruiting the practices and helping them with implementing our project into their clinic. [The facilitator is also] the engagement person. The practice facilitator could be comparable to the champion in that way by connecting our team to the specific clinics.” – Grantee 5, interviewee 1

“Engaging with practices and primary care providers to provide education, working with staff to implement screening, and constant communication, and following up and providing reminders.” - Grantee 4, interviewee 2

In year 2 we discovered that while most practice facilitators initially focused on recruitment, they quickly discovered that their roles extended far beyond this initial expectation. As the scope of their responsibilities widened, they began to engage in a broader range of supportive activities designed to enhance practice operations and outcomes, including the provision of incentives to encourage and sustain change. Throughout the project, facilitators continuously adapted their strategies and activities in response to the evolving needs and feedback of the participating practices. These iterative adjustments were made as challenges and opportunities emerged, allowing facilitators to refine their approach and better support sustained improvement at each site.

"The definition of practice facilitation had initially been different. When I started, practices really appreciated the external support — us coming in, setting up schedules, organizing the agenda, leading discussions, and posing questions. There's been a slight shift in attitude. I wouldn’t say we’re burdensome, but there's a sense of, 'Oh, we have to have another meeting with these guys'." - Grantee 3, interviewee 6

The evolving role of practice facilitators often brought about a shift in understanding and expectations, even among those directly involved in the process. This transformation was captured in the reflections of one grantee, who initially had a limited grasp of what the position entailed. Over time, however, their experience deepened their comprehension of the multifaceted nature of practice facilitation.

" I mean, admittedly, [when] I started, I wasn't sure what [a] practice facilitator meant. I [assumed] based [on] the words itself what it was, but I don't think I knew what it fully entailed. So, [now that] it's been [over] a year now, I think I have a different understanding of what it means to be a practice facilitator. [It's not just reaching out] to a practice and getting them to onboard, but really providing support, tools, and checking it in. [It's more than] what I thought was the definition of it." - Grantee 1, interviewee 1

### Theme 2: Supporting process improvement in clinical settings

Supporting process improvement in clinical settings is a key role of practice facilitators. Engagement typically began with practice leadership or the physician in charge to secure initial buy-in and set expectations. As implementation progressed, facilitators interacted with a range of practice staff—while other clinicians were sometimes involved, most ongoing support and communication centered on front desk personnel, office managers, and nurses or medical assistants. This broad-based engagement allowed facilitators to directly address the unique needs and workflows encountered at each site.

Practice facilitators frequently stressed the significance of providing resources that were genuinely useful and relevant to each practice. Rather than relying on standardized materials, facilitators prioritized tailoring support and tools to fit the distinctive requirements of each setting.

“So, it just depends on what's required of us to carry out what they need because that's what we're here for, to assist them, to make their job easier. Because we really need this partnership, and we really need their commitment to us to get the participants, and we'll help recruit and refer to see how beneficial the program can be.” – Grantee 5, interviewee 2

“What we're providing is a checklist… on what we heard from [the practice] that we think might be options that would work well. [We ask each practice] ‘Which of them are attractive? How would you want to modify it?’ [The resource checklist] goes generally into the bundles of screening, diagnosing, providing education, and offering treatment options. For each practice there is a curated [set of resources], based on what we heard and think might be helpful.” – Grantee 1, interviewee 1

Practice facilitators also reported the need for a strong commitment to continuous engagement and support for clinic sites to facilitate process changes and implementation. This ongoing interaction encompasses regular check-ins, email updates, and availability for assistance, providing both technical support and responsiveness to specific needs such as additional resources, training, or educational talks. It also includes systematic monitoring and communication that is clear and either structured or adaptive, to facilitate the adoption and implementation of new processes in clinics. Communication and outreach strategies varied across grantee projects, tailored to what proved effective for the practices they were engaging with (Table 3). However, within each project, there was consistency among facilitators regarding how communication and outreach was managed. Although the evaluation team did not systematically track contact frequency, qualitative data indicate that facilitators communicated with sites regularly—often several times per month—using emails, virtual meetings, phone calls, and in-person visits as needed. The frequency and mode of contact varied by site and project phase.

“Aside from my usual check-ins, I try to follow up during the week of the go-live, then again a week or a few weeks after, and sometimes even a few months later, just to make sure everything is going smoothly. I like to reaffirm my support by saying, "Hey, just checking in. Is everything okay? Remember, I'm here if anything goes wrong or if you have any recommendations. Don't hesitate to reach out." - Grantee 1, interviewee 1

**Table 3:** Communication Strategies Leveraged by Practice Facilitators

	**Grantee 1**	**Grantee 2**	**Grantee 3**	**Grantee 4**	**Grantee 5**
Tailored communication for impact awareness	✓	✓		✓	✓
Town halls	✓				✓
Leveraging networks for introductions	✓	✓		✓	✓
In person meetings		✓	✓	✓	✓
Video calls	✓	✓	✓		✓
Phone calls		✓	✓		✓
Personalized email communications	✓	✓	✓	✓	✓
Pre-recorded video		✓	✓		
Bilingual strategy	✓	✓			
Microsoft teams	✓				✓
WhatsApp					✓

We observed that practice facilitators highlighted the importance of setting realistic expectations for process change.

“Onboarding is practice facilitation. [when] I talk to these site champions, I tell them, change processes are slow. Anytime you try to change a process at a clinic, it takes time. So I look at it as we have six months to try to change a process at your clinic, and we're going to touch base a couple of times. I’m going to help you as much as I can. I'm always here if you need me. I do have practices that reach out through Teams messages and say, 'Hey, can we get some more brochures?' or 'Can you come talk to this group?' They will ask for things too." - Grantee 5, interviewee 3

Facilitators also recognized that simplifying processes and providing clear, actionable guidance were crucial in ensuring staff felt comfortable and confident in implementing new workflows. This approach not only facilitated smoother transitions but also empowered staff to engage more actively with the process improvements.

"And then we also wrote up a semi-simple workflow sheet document which was like, after you connect, do four things. I think we simplified it enough where staff felt a bit more comfortable with it. We did offer support visits, so after implementation and enrollment launch for these sites, we dropped off materials." - Grantee 4, interviewee 2

### Theme 3: Leveraging technology to facilitate change in healthcare practices

We found that while all practice facilitators utilized technology (electronic health records (EHR), patient portals, mobile health applications, digital communication platforms such as Microsoft Teams, tele-education platforms like Project ECHO, and data dashboards), they did not all use the same types of technology (Table 4). In year two, practice facilitators reported increasingly harnessing technology to support healthcare practices and drive organizational change, offering hands-on support to enhance these efforts. This approach includes the development and integration of various technological tools designed to streamline operations, improve patient care, and facilitate meaningful interactions between providers and patients.

"So the SharePoint site has a data dashboard that everybody is always usually excited about seeing because it's fun. But I'll be honest, I don't know that it's in use a whole lot as far as bringing more awareness to the program. We end up using it for like a measurement stick on how well the practices [are] increasing their numbers as far as getting women identified and diagnosed." - Grantee 5, interviewee 3

**Table 4:** Technology Leveraged by Facilitators

	**Grantee 1**	**Grantee 2**	**Grantee 3**	**Grantee 5**	**Grantee 4**
Technological and informational resources (e.g., brochures, online videos)	✓	✓	✓	✓	
Electronic Health Records (and Enhancements)	✓	✓	✓	✓	✓
Patient care app	✓			✓	✓

Even though practice facilitators recognized the benefits of integrating technology tools, they still faced challenges with the initial complexity of these tools, and had to navigate the use of different tools across projects and within practices, rather than relying on a single, unified solution.

"So we're using [specific EHR], which is just kind of a pain at first, and starting to use it for one benefit at this point. And we also have our care pathway...which is what our nurse navigators use to educate our patients and offer treatments and lifestyle behavioral changes." - Grantee 4, interviewee 2

Practice facilitators praised the development of user-friendly, sustainable tools, such as a revised care pathway in EHRs and healthcare management application. This highlights the commitment to integrating technology as a core component of long-term healthcare improvement strategies. Practice facilitators reported on ensuring the effectiveness of clinical decision support tools being integrated into the Electronic Medical Records (EMR) during the study. A specific highlight is the "best practice alert" tool, which has shown promising results in increasing the likelihood of provider-patient conversations about relevant health issues during visits. This tool activates when a provider opens a patient's chart, prompting immediate attention to specific patient concerns and recommending actions such as screening, providing patient education materials, suggesting appropriate non-pharmacologic therapies or behavioral interventions, and identifying medications to prescribe when indicated. However, clinicians reported that this tool posed a challenge, as they felt constrained by its workflow, which did not accommodate the needs of all patients.

"Well, I think some of the clinical decision support tools are really cool. One is our best practice alert [a real-time, automated notification within an EHR system that prompts healthcare providers to take specific actions based on evidence-based guidelines and patient-specific information] actually made a big difference. When we did our pilot at theaters, we told the providers, ‘when your patient screens positive, please talk to them.’ We emailed and then reached out on [a specific EHR] but then when we started the study [and used] best practice alert where the provider opens the chart when the patient's there and then it says, ‘please address and continue’." - Grantee 2, interviewee 1

### Theme 4: Barriers to practice facilitation

Practice facilitators reported barriers encountered in their efforts to effectively implement and manage the MUI interventions across various clinical settings. In establishing or recruiting practices, practice facilitators encountered several obstacles such as finding champions, convincing practices of the project's low burden, managing bureaucracy, and limitations of study.

#### Identifying champions

Practice facilitators emphasized that identifying champions within healthcare practices presented several barriers. While champions could be project managers, clinicians, office managers, or nurses, nurses most often stepped into this role due to their direct patient interactions and adaptability. In contrast, medical doctors and specialists were less frequently engaged because of heavier workloads and competing priorities. This discrepancy underscores the challenge of securing provider involvement, which is crucial for the success of practice facilitation efforts. Although nurses are not typically responsible for making final clinical management decisions, their role as champions is crucial in operationalizing workflow changes, implementing new processes (such as screening and education), and bridging communication between practice facilitators, providers, and staff. Despite these barriers, the involvement of champions—regardless of their professional background—has been pivotal to the implementation and sustainability of improvements.

“Getting a site champion that understands the need or has some kind of compassion for it is sometimes a barrier. I've had to change site champions before because I just could not get a hold of them and I don't know why...the ones that are engaged are the ones that you know are usually with the patients. I think mainly just time and availability are the two biggest barriers in making sure that we've got a site that cares enough……The nursing staff is the typical site champion." - Grantee 5, interviewee 3

#### Convincing practices of the project's low burden

Another reported challenge in engaging practices is often arranging that initial meeting, as this sets the stage for explaining the project's demands in a manner that is manageable for practices already burdened by their existing workload.

“The biggest challenge is probably getting that first meeting set up, because once you have that first meeting set up, it's really much smoother to explain what's being asked and kind of explain it in a more digestible way for practices that are already overworked and overwhelmed. [We] market [the] project as being low burden to the practices. - Grantee 4, interviewee 1

#### Navigating bureaucracy

Another key challenge was the importance of navigating healthcare institutional bureaucracy as part of the practice facilitation role. Practice facilitators reported that practices, regardless of size, adhere to a formal structure that entails specific rules and procedures that must be followed to implement any changes to the practices’ standard operating procedures. Consequently, convincing each practice to participate in the interventions necessitated that each facilitator navigate the varying levels of bureaucracy.

“I think we're having a little bit of an issue getting through some of that red tape. There definitely is a hierarchy pecking order and getting the blessing of the person that's overseeing this, it took a while to get in and get that blessing and then it was like, well, can we do this.” – Grantee 5, interviewee 2

Some practice facilitation approaches had to adapt to various bureaucratic layers such as acknowledging hierarchical structures of approval. Depending on the site and the grantee intervention, there were various processes that needed to be followed and stamps of approval that needed to be provided prior to sites being recruited or the intervention getting started.

“The thing is, it was really hard - the bottleneck, because we can't go and talk to all the sites until we get approval from the medical director. So, getting their [Medical Director(s)] green lights was a little difficult. There was one medical director that we that we had a meeting with who was not supportive of the project. Therefore, we could not approach her sites to do this. When the medical director is behind this project that can really help, especially, if the medical director is talking to their site.” – Grantee 4, interviewee 2

We observed that these bureaucratic challenges are not only initial hurdles but also persist throughout the implementation process.

“I guess you'd say [the] most barriers is us getting in there. Then after that there are administrative barriers to implementing because there's a bunch of committees that decide what should be interruptive alerts or what should be screening.” – Grantee 2, interviewee 2

Practice facilitators also learned that different health systems have their own unique procedures and bureaucratic process that are specific to their size and structure. Recognizing these differences was important to effectively navigate the systems and achieve desired outcomes for project implementation.

“I think the biggest thing that I've noticed is that the clinics that are part of a large health system have a lot more trouble committing to the project than those who are independent practices or smaller health systems…big healthcare systems have more layers of folks who need to approve it. The smaller practices are more nimble and have more autonomy to choose to participate.” – Grantee 3, interviewee 2

#### Limitations of study

Further, the challenges faced by practice facilitators is reported as often being compounded by the structural limitations of their studies and the practical constraints within healthcare practices. These limitations reportedly can prevent facilitators from addressing certain systemic issues or provide the level of support that practices might need.

“ With the way our study is structured, we're only providing [some practices with] additional support and connecting clinics with existing community resources. One clinic had [an issue with] insurance coverage. Asking about a problem during a physical and treating it adds an extra charge. The main requests have been for systemic changes with insurance, which we unfortunately can't address, or [for better] location access” - Grantee 3, interviewee 4

The practice didn't have time to do [the sessions]..."No one takes it [additional orientation sessions], and I think [it's because of] bandwidth and time. Even when additional support, like training, is offered, it's usually not needed or wanted." - Grantee 1, interviewee

### Theme 5: Sustainable practice changes beyond practice facilitation

As practice facilitation nears completion in the MUI initiative, facilitators are strategically working to ensure that the impact of their efforts is sustained well beyond the immediate study period. These strategies include fostering clinic autonomy, revisiting providers to collect valuable feedback on the effectiveness of interventions, providing training, ensuring resource availability, promoting the use of clinical decision support tools developed during the study, and finalizing patient recruitment and follow-up surveys.

"We began planting seeds for conversations they could continue on their own, which is ultimately the goal. As the project has progressed and clinics have begun to take more ownership, which is what we aim for…However, my role has remained consistent, I’m here to provide support. I still schedule and lead these meetings with the intention of facilitating and sparking these initial discussions. Whether or not they take ownership and run with it is up to them" - Grantee 3, interviewee 6

Some practice facilitators are transitioning from heavily guided facilitation to encouraging clinic autonomy by ensuring ownership of the project and using train the trainer model.

“[A few times], the practice manager [asked], 'Can you provide an orientation for our rooming team or for the Clinical Operations Coordinator (COC)?' [The COC works closely with] the rooming team. [If I provide that individual orientation], they're well aware of the workflow and can support the rooming team if needed." - Grantee 1, interviewee 1

Moreover, some practice facilitators plan to extend the reach of clinical decision support tools developed for the study to the study control groups and other providers who did not participate in the intervention. The intention is to make these tools more visible and accessible in the EMR system. Although they are currently available, they are not prominently used. Practice facilitators are intentional about a follow-up phase that involves educating providers about these tools and encouraging their use in clinical practice, particularly for managing urinary incontinence.

“We plan to share all the clinical decision support tools in our control groups cause [they didn’t] get the intervention. I think my plan would be once the study is completed to go back and share at least the support tools that were built in the electronic medical record. At the moment they're not restricted to certain physicians, but if you don't know to look for it, you're not going to see it” - Grantee 2, interviewee 1

## Discussion

This study builds on a growing body of evidence for the value of practice facilitation in improving the adoption of evidence-based care in primary care, addressing gaps around the evolving and diverse roles of practice facilitators in UI care, including their adaptability, strategies for sustaining improvements, and integration of digital tools. Our qualitative, multi-site evaluation extends prior work [21, 22, 41] by illuminating how practice facilitation operates in dynamic, real-world environments, highlighting what it takes to move from initial engagement to embedded, sustainability effort. These insights are especially timely given the persistently high burden of UI among older women in the U.S. and the recognized gap between effective interventions and routine clinical practice [25–29]. To our knowledge, this represents the first published study to utilize practice facilitation specifically for the management of UI in primary care, offering novel and in-depth perspectives on the challenges and opportunities for integrating evidence-based UI care in everyday practice.

A central observation from our findings is the evolving nature of the practice facilitator role throughout the course of the projects. Facilitators initially focused on practice recruitment and early engagement; however, as trust with clinical teams grew and project scope expanded, facilitators took on increasingly central roles in workflow redesign, providing ongoing technical assistance, and even offering incentives to sustain new behaviors. This evolution mirrors the literature’s recognition that practice facilitation is not a static occupation but a highly adaptive process, requiring flexibility as work priorities shift in response to emerging needs within practices [7]. Capturing this role evolution through repeat interviews highlights the necessity for facilitator training programs to focus not just on quality improvement techniques, but also on the skills needed for adaptive leadership and continuous relationship management.

Another key finding concerns the bureaucratic and organizational barriers that facilitators regularly encounter. Across diverse sites, facilitators reported the need to skillfully navigate complex and hierarchical approval processes, which often differ significantly by system, practice size, or local context. Understanding—and adjusting to—these institutional structures is essential for gaining buy-in and implementing changes, as highlighted in prior studies on facilitation barriers and sustainment [23]. This is important because studies have shown that practice facilitator with leadership buy-in for instance, achieve higher implementation quality of evidence-based programs [10]. Our study surfaces practical strategies facilitators deployed, from identifying appropriate champions to incrementally adapting interventions for local context, further enriching the literature by describing granular, situation-specific approaches to common challenges.

Sustainability continues to be a critical concern in practice facilitation research, given the risk of new practices fading once external support is withdrawn [8, 42]. Our study demonstrates that facilitators ought to proactively foster clinic autonomy, promote train-the-trainer approaches, and leverage technology—particularly clinical decision support tools within the EHR—to help ensure that improvements are not only adopted but maintained. These findings align with and extend the view that strategies tailored to specific clinical and organizational environments enhance sustainability, reinforcing the imperative to “work with the grain” of each unique practice [7].

We also found that practice facilitators are at the forefront of integrating digital tools—such as telehealth, EHR-based decision support, and patient portals—into quality improvement work, addressing a previously underexplored area of the literature [7]. While facilitators were generally enthusiastic about the promise of technology for streamlining practice change, they also identified challenges with integration, adoption, and real-world usability. This gap between technological potential and daily utility underlines the need for facilitator involvement in tool selection, adaptation, and ongoing feedback to ensure digital strategies are practical and sustainable for the clinics they serve.

Despite many successes, persistent challenges remain. Facilitators described ongoing difficulties in securing physician champions, whose clinical commitment is vital to embedding quality improvement into routine workflows as competing priorities and heavy workloads often limited provider engagement. Although nurses frequently filled this role, further strategies are needed to boost provider buy-in and participation, consistent with prior findings on facilitator-physician relationships. Moreover, arranging initial meetings and communicating the low burden of participation proved significant hurdles in overwhelmed practices, necessitating approaches that minimize additional demands while conveying clear value. Another factor that may have contributed to these challenges is that only one of the grantee sites provided formal training to their facilitators, potentially impacting their preparedness to navigate complex barriers and engage practices effectively. Studies show that facilitators may benefit from additional training to help with their recruitment and engagement of practices [21, 22]. Finally, facilitators noted systemic constraints—including insurance coverage and resource limitations—that remain outside their immediate influence, emphasizing the need for broader organizational and policy interventions to bolster quality improvement adoption. While this study contributed to addressing the gap in the literature regarding repeated measure studies of practice facilitation [8], we recommend that future research focus on conducting more longitudinal studies to observe the long-term impact of practice facilitation.

### Implications for research and practice

This study advances the literature by offering rare, longitudinal qualitative insights into the lived realities of practice facilitation across multiple settings and project phases for UI. Our repeat interviews capture both the adaptability required of successful facilitators and the central challenge of sustainability in quality improvement interventions. The results reinforce the importance of (1) robust, flexible facilitator training, (2) strategies for navigating organizational structures, (3) tailored approaches to digital tool integration, and (4) explicit plans for building clinic-level autonomy to ensure long-term practice change.

In addition to these factors, our findings further underscore the critical role of early and ongoing engagement with medical directors and leadership when implementing new quality improvement initiatives. Establishing direct dialogue with key leaders, involving them in the planning process, clearly communicating both the benefits and minimal burden of the intervention, and leveraging peer success stories to build trust and buy-in are all strategies that can facilitate leadership support. It is also important to align projects with organizational priorities and maintain transparent, open communication throughout. Together, these efforts are likely to improve buy-in from leadership, which is essential for smooth implementation and sustainability.

While our work addresses the gap in repeated measures of practice facilitation [8, 21], more sustained, prospective research is needed to follow the long-term outcomes of both facilitators and the practices they support. Future studies should focus on measuring the durability of quality improvement gains, the scalability of facilitator-driven strategies, and the ongoing integration of digital tools—particularly in under-resourced and high-burden settings.

### Limitations

These limitations pertain both to our evaluation methods and, in many cases, to the real-world challenges faced by the initiative itself, highlighting areas for both improved study design and implementation in future projects. This study has several limitations related to the design and scope of our evaluation that may affect interpretation and generalizability of the findings. The relatively small number of practice facilitators and grantee projects inherently limits the extent to which results can be applied to other settings, and the diverse characteristics of participating sites—including academic, tribal, urban, rural, and federally qualified health centers—mean that implementation strategies and outcomes are not always directly comparable. Additionally, our qualitative interview data may be subject to response bias, as anonymity could not be guaranteed for participating practice facilitators. Although interviews were conducted confidentially by an independent evaluation team, the absence of an anonymous feedback option may have influenced the candor of facilitator responses. Future research would benefit from incorporating anonymous survey methods and recruiting a larger, more diverse sample of facilitators and sites to enhance the robustness and external validity of the findings.

A number of important implementation limitations within the initiative itself also affected our results. Notably, only one grantee provided formal, structured training to practice facilitators, while the majority of the practice facilitators relied on on-the-job learning and did not possess specific facilitation credentials prior to this role. This variation in experience and preparation may have led to inconsistencies in facilitator effectiveness, especially during the initial engagement phase. For instance, physicians who served as facilitators contributed valuable clinical credibility and workflow insight, supporting clinician engagement, but often faced time constraints that limited their ability to focus on facilitation activities.

Significant challenges were also encountered in arranging introductory meetings with practices, as sites faced unique hurdles related to leadership buy-in, competing demands, and resource constraints. The content and process of these meetings, as well as the materials used, were not standardized, potentially resulting in uneven communication and expectations across sites. Furthermore, variability in the identification and responsibilities of practice champions across sites may have contributed to inconsistency in implementation. While this flexibility allowed adaptation to local context, a lack of standardization in the champion role complicates the comparison of results across different practices and may limit generalizability.

## Conclusion

Practice facilitators are central to bridging the gap between evidence and practice in complex clinical environments. Our findings demonstrate both the promise and ongoing challenges of facilitation in primary care UI management. By elucidating the diverse, evolving roles of facilitators and surfacing new strategies for overcoming barriers and sustaining improvements, this study informs the next generation of facilitation-based quality improvement initiatives for under-addressed and high-priority conditions in primary care.

## Supplementary Information

Supplementary Material 1.

## Data Availability

Data is available with approval from the Agency of Healthcare Research and Quality.
